# A gene expression signature associated with survival in metastatic melanoma

**DOI:** 10.1186/1479-5876-4-50

**Published:** 2006-11-27

**Authors:** Susanna Mandruzzato, Andrea Callegaro, Gianluca Turcatel, Samuela Francescato, Maria C Montesco, Vanna Chiarion-Sileni, Simone Mocellin, Carlo R Rossi, Silvio Bicciato, Ena Wang, Francesco M Marincola, Paola Zanovello

**Affiliations:** 1 Oncology Section, Department of Oncological and Surgical Sciences, University of Padova, Padova, Italy; 2 Department of Chemical Process Engineering, University of Padova, Padova, Italy; 3 Pathology Section, Department of Oncological and Surgical Sciences, University of Padova, Padova, Italy; 4 Istituto Oncologico Veneto, Padova, Italy; 5 Surgery Section, Department of Oncological and Surgical Sciences, University of Padova, Padova, Italy; 6 Department of Transfusion Medicine, Clinical Center, NIH, Bethesda MD, USA

## Abstract

**Background:**

Current clinical and histopathological criteria used to define the prognosis of melanoma patients are inadequate for accurate prediction of clinical outcome. We investigated whether genome screening by means of high-throughput gene microarray might provide clinically useful information on patient survival.

**Methods:**

Forty-three tumor tissues from 38 patients with stage III and stage IV melanoma were profiled with a 17,500 element cDNA microarray. Expression data were analyzed using significance analysis of microarrays (SAM) to identify genes associated with patient survival, and supervised principal components (SPC) to determine survival prediction.

**Results:**

SAM analysis revealed a set of 80 probes, corresponding to 70 genes, associated with survival, i.e. 45 probes characterizing longer and 35 shorter survival times, respectively. These transcripts were included in a survival prediction model designed using SPC and cross-validation which allowed identifying 30 predicting probes out of the 80 associated with survival.

**Conclusion:**

The longer-survival group of genes included those expressed in immune cells, both innate and acquired, confirming the interplay between immunological mechanisms and the natural history of melanoma. Genes linked to immune cells were totally lacking in the poor-survival group, which was instead associated with a number of genes related to highly proliferative and invasive tumor cells.

## Background

Although cutaneous melanoma is a relatively rare tumor, its incidence is rising sharply, with minimal progress made in its treatment [1]. When lymph node or distant metastases are present, clinical outcome is poor but highly variable. In fact, patients in the same TNM stage can have very different clinical outcomes, which is particularly true for TNM stage III. Currently, the most useful prognostic factors in metastatic disease are the metastatic site (e.g. subcutaneous vs visceral localization) and lactate dehyrogenase (LDH) plasma levels [2-4].

Despite significant efforts to identify independent predictors of melanoma outcome, no generally accepted histopathological or molecular marker defines disease subsets with clinically different outcomes [5-7]. Understanding differences in clinical behavior is important not only in the design and analysis of clinical trials, but also in planning different therapeutic strategies, such as adjuvant treatment.

In recent years, microarray technology has been extensively used in cancer research to obtain gene expression profiles aimed at identifying tumor classes, disease-related genes and new markers for predicting clinical outcome. Several studies also demonstrated that gene expression signatures can be used as a tool to predict survival of cancer patients [8-11]. As regards melanoma, analyses of gene expression have shed new light on the progression from local to metastatic disease as well as on melanoma immune responsiveness [12-14].

In this study, we correlated the gene-expression profile of tumors with overall survival in a cohort of patients with stage III and IV melanoma to determine whether survival among patients is reflected by specific sets of expressed genes. By using the significance analysis of microarrays (SAM), we identified 80 differentially expressed probes. We also generated a survival prediction model that resulted in 30 survival-related probes, all included in the set identified by SAM.

## Methods

### Patients and tissue collection

From 1997 to 2000, we collected 43 fresh metastatic melanoma biopsies from 38 patients with stage III and IV melanoma who underwent surgery as a part of the diagnostic work-up or therapeutic strategy. For three of these patients, we collected two biopsies, and in one case we obtained three biopsies. Immediately after surgery, half specimen was fixed in formalin and processed for routine histology, and the other half was snap-frozen in liquid nitrogen and stored at -80°C until use for RNA extraction. This study was examined and approved by the Ethics Committee of the local Health and Social Services (Azienda Ospedaliera, Padova) in accordance with the ethical standards laid down in the 1964 Declaration of Helsinki.

### RNA extraction, amplification and labeling

Total RNA was extracted from frozen material by homogenizing the sample in TRIZOL reagent (Invitrogen, CA), according to the manufacturer's instructions, amplified into anti-sense RNA (aRNA) and reverse transcribed into fluorescent-labeled cDNA for hybridization to a custom-made 17,500-gene cDNA-based array as previously described [15,16]. After amplification, the quality of aRNA was tested with the Agilent Bioanalyzer 2000 (Agilent Technologies, Palo Alto, CA). Total RNA from peripheral blood mononuclear cells pooled from 6 normal donors was extracted and amplified to prepare reference aRNA to be cohybridized in all experiments with test aRNA. cDNA targets were labeled with Cy3 for reference material and Cy5 for test material. The 32 × 24 × 23 (17,500 spots) human cDNA microarray was prepared in the Immunogenetics Section of Transfusion Medicine, Clinical Center, National Institute of Health, Bethesda. Clones for printing of 17 k cDNA array included a combination from a RG_HsKG_031901 7 k clone set and 10,000 clones from the RG_Hs_seq_ver_070700 40 k clone set (Research Genetics, Huntsville, AL). The cDNA clones include 12,072 uniquely named genes, 875 duplicates of named genes; the remainder consisted of expression sequence tags (complete gene list and printing layout are available [17]. The hybridization was carried out at 65°C for 16–18 hours, and the slides were then washed and scanned on a Gene Pix 4000 scanner at variable PMT to obtain optimized signal intensities with minimum (< 1% spots) intensity saturation.

### Statistical analysis

Before statistical analysis, data were loaded and filtered using the software package BRB Array Tools [18]. Specifically, a statistical significance criterion based on the variance was used to filter log expression variations and remove those genes whose expression log ratios did not differ significantly from the median value (p-value ≤ 0.01). Genes were also excluded if the percentage of missing expression values was greater than 50%, resulting in a total of 4,845 probes used for further analysis.

Significance analysis of microarrays (SAM), [19,20] was applied to identify genes correlated with patient overall survival. Briefly, SAM computes a score for each gene that measures the strength of transcript correlation with survival. This score is the maximum-likelihood score statistic from Cox's proportional hazards model (Cox score). A threshold value was chosen to give a reasonably low false positive rate, as estimated by repeatedly permuting the survival times and counting the number of genes that were significant at each threshold. Missing data were handled using the K-nearest neighbors imputer (k = 10) of the SAM imputation engine.

Survival prediction was calculated using supervised principal components (SPC)[21]. This analysis is similar to conventional principal component analysis although SPC uses a subset of predictors that are selected based on their association with outcome (see additional file 1 for a detailed description).

SPC were applied using the R *superpc *package [22].

### Immunohistochemistry

The following antibodies were used: HLA Class II (DR) (Clone LN-3, Novocastra, Newcastle upon Tyne, UK, dilution 1:50) and IL-4R (clone 25463, R&D Systems, Abingdon, UK).

Tissue sections of 4-μm were cut from formalin fixed, paraffin-embedded tissue blocks and rehydrated. Endogenous peroxidase activity was blocked with incubation of the slides in 3% H_2_0_2 _in methanol and then, primary antibodies were applied at room temperature for 45 minutes. Binding sites of the primary antibodies were visualized using the Envision Plus detection System (Dako, Glostrup, Denmark) for HLA-DR and the Vectstain ABC kit (Vector laboratories, Burlingame, USA) for IL-4R.

Sections were developed using 3-amino-9-ethylcarbazole (Biogenex, San Ramon, USA), the slides were counterstained with hematoxylin and mounted in aqueous medium.

Immunostaining for IL4R and HLA-DR was separately evaluated in tumor cells and in intratumoral and peritumoral inflammatory cells and scored semi-quantitatively as 0 (no staining), 1+ (0–25%), 2+ (26–50%), 3+ (> 50%).

## Results

### Patient Characteristics

The clinical and pathological characteristics of the 38 melanoma patients (20 females and 18 males) are reported in Table 1. The median age at diagnosis was 58 years (range 23–82). Twenty-two (58%) patients had stage III and 16 (42%) stage IV melanoma at the time of biopsy. Two stage IV patients with subcutaneous and lung metastases respectively were also affected by ocular melanoma. Before biopsy, thirteen patients had been treated: adjuvant interferon (adIFN) for 7 patients; systemic chemotherapy (Sy-CT) for 2 patients; Sy-Ct and adIFN in 1 case; isolated hyperthermic perfusion (HIP) in 1 case; adIFN, Sy-CT and HIP in 1 case; and Sy-CT and radiotherapy in another patient. After a median follow-up of 65 months (range 48–103), 9 patients (7 females and 2 males) were still alive without clinical evidence of disease. Twenty-nine patients (76%) died as a consequence of the melanoma progression; 7 of whom of brain metastases (24%).

**Table 1:** Clinical characteristics of the 38 patients with melanoma considered in the present study.

**Characteristic**	**Patients (N = 38)**	
**Gender**	#	%

Male	18	47
Female	20	53
**Age**		
Median	58	
Range	23–82	
Stage III*	22	58
Stage IV*	16	42
Previous Treatment**		
No	25	66
Yes**	13	34
Interferon***	9	69
Chemotherapy	5	38
Isolated Perfusion	2	15
Radiotherapy	1	7

* According the new AJCC staging system for melanoma [3]

** Some patients received more than one treatment

*** Alone or in combination with chemotherapy

### Identification of a gene set correlated with survival

**Figure 1:**
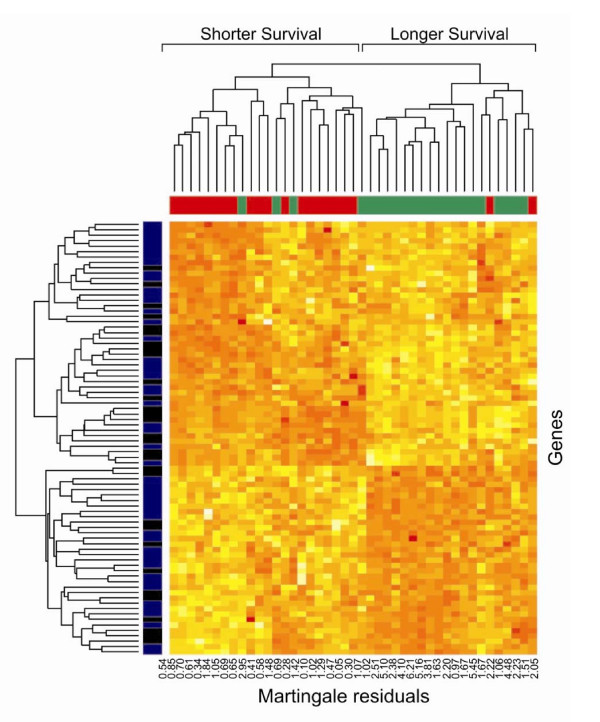
**Supervised hierarchical representation of the 80 probes selected by SAM and related to survival probability**. In blue the 80 probes selected by SAM and in black the 30 transcripts used to build the SPC survival predictor. Samples are labeled according to survival probability curves of Figure 2 (solid and dotted line for shorter and longer survival, respectively) and Martingale residuals of the null model are reported for each specimen. The Martingale residuals represent the difference between the observed and expected number of events for each individual. Thus, large negative residuals indicate that the observed number of deaths is less than the expected number of deaths, i.e. the individual lived longer than expected. Large positive residuals (closed to 1) indicate that the observed number of deaths is greater than the expected number of deaths, i.e. the individual's survival time was shorter than expected.

Using nucleotide arrays, we generated gene-expression profiles for 43 metastatic melanomas by analyzing 30 metastatic lymph-nodes, 11 cutaneous, 1 lung and 1 gastrointestinal metastases [15,16]. To identify a gene expression profile correlated with overall survival, we used SAM class comparison. This method identified a total of 80 probes (Tables 2 and 3) corresponding to 70 unique genes associated with survival, 45 negatively (Table 2) and 35 positively (Table 3), with a median of 13.06 false positive (q-value < 15%) selecting Δ = 0.36 and 500 random permutations. The value of Δ was selected as a compromise between the total number of significant probes and the false positive rate. When the score is negative, higher expression correlates with longer survival, whereas a positive score indicates that higher expression correlates with shorter survival. The expression pattern of the 80 probes identified by SAM as related to survival in the 43 samples is shown in the dendrogram of Fig. 1. In this analysis stage III and stage IV patients are equally distributed among the 2 groups with significantly different survival times (data not shown), clearly indicating that our study group did not have a selection bias.

**Table 2:** Top 45probes whose increase in expression is significantly associated with longer survival.

**Feature ID**	**Gene symbol**	**Gene name**
IMAGE:128126	DAF	Decay accelerating factor for complement
IMAGE:504226	CD53	CD53 antigen
IMAGE:66560	IGLC2	immunoglobulin lambda locus
IMAGE:770670	TNFAIP3	Tumor necrosis factor, alpha-induced protein 3
IMAGE:153411	HLA-DRA	Major histocompatibility complex, class II, DR alpha
IMAGE:767183	HCLS1	Haematopoietic cell-specific Lyn substrate 1
IMAGE:344134	IGLL1	Immunoglobulin lambda-like polypeptide 1
IMAGE:2448698	HLA-DRB4	major histocompatibility complex, class II, DR beta 4
IMAGE:2443695	HLA-DRB4	major histocompatibility complex, class II, DR beta 4
IMAGE:897641	HNRPU	Heterogeneous nuclear ribonucleoprotein U
IMAGE:210921	NFKBIZ	Nuclear factor of kappa light polypeptide gene enhancer in B-cells inhibitor, zeta
IMAGE:141806	TNFAIP3	Tumor necrosis factor, alpha-induced protein 3
IMAGE:1572582	NCF4	Neutrophil cytosolic factor 4, 40 kDa
IMAGE:2508563	TNFAIP3	Tumor necrosis factor, alpha-induced protein 3
P48460	LTB	lymphotoxin beta (TNF superfamily, member 3)
IMAGE:714453	IL4R	IL-4 receptor alpha chain
IMAGE:1469292	PIM2	Pim-2 oncogene
IMAGE:2443532	SVIL	Supervillin
IMAGE:1585327	AXIN2	Axin 2 (conductin, axil)
IMAGE:814802		Similar to serine/threonine kinase 4
IMAGE:2512080	CST1	Cystatin SN
IMAGE:827132	RAC2	Ras-related C3 otulinum toxin substrate 2
IMAGE:309864	JUNB	Jun B proto-oncogene
IMAGE:293925	LYZ	Lysozyme
IMAGE:789314	CD1D	CD1D antigen, d polypeptide
IMAGE:246748	BTK	Bruton agammaglobulinemia tyrosine kinase
IMAGE:295868	LAPTM5	Lysosomal associated multispanning membrane protein 5
IMAGE:199334	C14orf117	chromosome 14 open reading frame 117
IMAGE:50877	LOC91316	similar to bK246H3.1 (immunoglobulin lambda-like polypeptide 1, pre-B-cell specific)
IMAGE:346860	SOD2	Superoxide dismutase 2, mitochondrial
IMAGE:727988	CDW52	CD52 antigen
IMAGE:320393	HLA-DQA1	Major histocompatibility complex, class II, DQ alpha 1
IMAGE:2010319	NALP1	NACHT, leucine rich repeat and PYD (pyrin domain) containing 1
IMAGE:243741	UBD	Ubiquitin D
IMAGE:868368	TMSB4X	Thymosin, beta 4, X-linked
IMAGE:1552744	CD2	CD2 antigen (p50)
IMAGE:855547	HLA-DRB1	major histocompatibility complex, class II, DR beta 1
IMAGE:342378	DUSP5	Dual specificity phosphatase 5
IMAGE:823779	PLEK	Pleckstrin
IMAGE:2562146	MAPK13	Mitogen-activated protein kinase 13
IMAGE:682418	ELF4	E74-like factor 4
IMAGE:712209	ARHGAP15	Rho GTPase activating protein 15
IMAGE:770014	TRA@	T cell receptor alpha chain
IMAGE:2281706	ITK	IL2-inducible T-cell kinase
IMAGE:428412	GZMK	Granzyme K

**Table 3:** Top 35 probes whose increase in expression is significantly associated with shorter survival.

**Feature ID**	**Gene symbol**	**Gene name**
IMAGE:809557	MCM3	MCM3 minichromosome maintenance deficient 3
IMAGE:823859	GJB2	Gap junction protein, beta 2, 26 kDa (connexin 26)
IMAGE:505573	PYGL	Phosphorylase, glycogen; liver
IMAGE:626544	MYO10	Myosin X
IMAGE:2488304	TRIM32	Tripartite motif-containing 32
IMAGE:200402	C20orf129	Chromosome 20 open reading frame 129
IMAGE:269791	DCT	Dopachrome tautomerase (tyrosine-related protein 2)
IMAGE:2297394	EFHD1	EF-hand domain family, member D1
P07338	CSPG4	Chondroitin sulfate proteoglycan 4 (melanoma-associated)
IMAGE:897563	M-RIP	Myosin phosphatase-Rho interacting protein
IMAGE:504461	KMO&OPN3*	Kynurenine 3-monooxygenase & Opsin 3*
IMAGE:768571	SOX8	SRY (sex determining region Y)-box 8
IMAGE:138601	KIAA1026	Kazrin
IMAGE:626544	MYO10	Myosin X
IMAGE:1461664	BCHE	Butyrylcholinesterase
IMAGE:365826	GAS1	Growth arrest-specific 1
IMAGE:486175	SLC16A1	Solute carrier family 16, member 1
IMAGE:823886	MYH10	Myosin, heavy polypeptide 10, non-muscle
IMAGE:866882	FDFT1	Farnesyl-diphosphate farnesyltransferase 1
IMAGE:592802	RGS12	Regulator of G-protein signalling 12
IMAGE:815151	PRKCE	Protein kinase C, epsilon
IMAGE:2049072	ADAMTS5	A disintegrin-like and metalloprotease with thrombospondin type 1 motif, 5
IMAGE:811562	NEO1	Neogenin homolog 1 (chicken)
IMAGE:241705	PYGL	Phosphorylase, glycogen; liver
IMAGE:143919	CHEK1	CHK1 checkpoint homolog
IMAGE:811562	NEO1	Neogenin homolog 1 (chicken)
IMAGE:503889	P15RS	Hypothetical protein FLJ10656
IMAGE:504461	KMO&OPN3*	Kynurenine 3-monooxygenase & Opsin 3*
IMAGE:266263		cDNA FLJ31683 fis
IMAGE:269733	BNC2	Basonuclin 2
IMAGE:486561	PARVA	Parvin, alpha
IMAGE:453599	C14orf109	Chromosome 14 open reading frame 109
IMAGE:38925	EST	EST, Weakly similar to S10889 proline-rich protein
IMAGE:824776	DHFR	Dihydrofolate reductase
IMAGE:2018084	STK39	Serine threonine kinase 39

*Clone IMAGE:504461 maps to 2 Unigene Clusters

**Figure 2:**
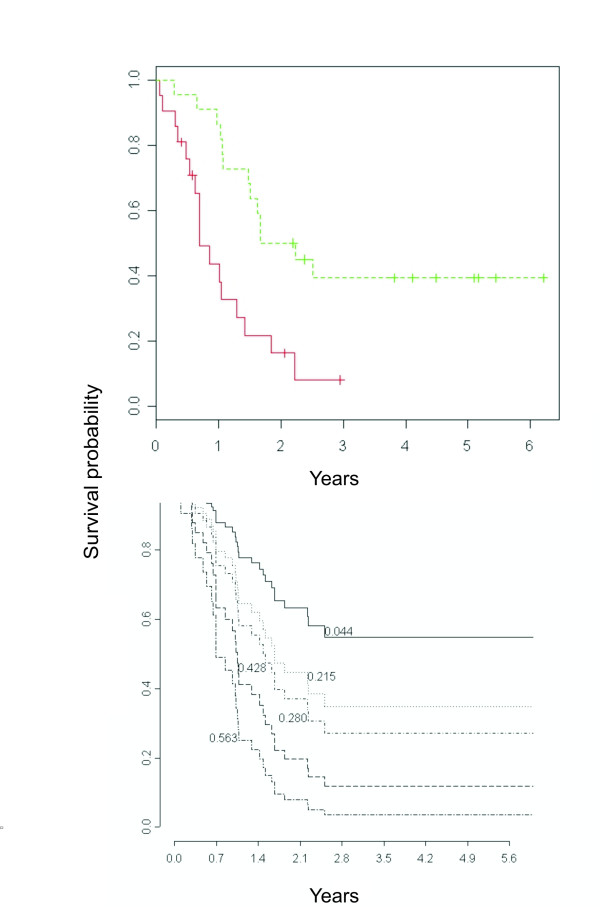
**Survival probability of the 43 samples**. A) Prediction was based on the v^1
 MathType@MTEF@5@5@+=feaafiart1ev1aaatCvAUfKttLearuWrP9MDH5MBPbIqV92AaeXatLxBI9gBaebbnrfifHhDYfgasaacH8akY=wiFfYdH8Gipec8Eeeu0xXdbba9frFj0=OqFfea0dXdd9vqai=hGuQ8kuc9pgc9s8qqaq=dirpe0xb9q8qiLsFr0=vr0=vr0dc8meaabaqaciaacaGaaeqabaqabeGadaaakeaacuWG2bGDgaqcamaaBaaaleaacqaIXaqmaeqaaaaa@2F4D@ model using 30 transcripts with *θ *= 1.7 (v^130
 MathType@MTEF@5@5@+=feaafiart1ev1aaatCvAUfKttLearuWrP9MDH5MBPbIqV92AaeXatLxBI9gBaebbnrfifHhDYfgasaacH8akY=wiFfYdH8Gipec8Eeeu0xXdbba9frFj0=OqFfea0dXdd9vqai=hGuQ8kuc9pgc9s8qqaq=dirpe0xb9q8qiLsFr0=vr0=vr0dc8meaabaqaciaacaGaaeqabaqabeGadaaakeaacuWG2bGDgaqcamaaDaaaleaacqaIXaqmaeaacqaIZaWmcqaIWaamaaaaaa@3130@). Dotted and solid curves represent samples with v^130
 MathType@MTEF@5@5@+=feaafiart1ev1aaatCvAUfKttLearuWrP9MDH5MBPbIqV92AaeXatLxBI9gBaebbnrfifHhDYfgasaacH8akY=wiFfYdH8Gipec8Eeeu0xXdbba9frFj0=OqFfea0dXdd9vqai=hGuQ8kuc9pgc9s8qqaq=dirpe0xb9q8qiLsFr0=vr0=vr0dc8meaabaqaciaacaGaaeqabaqabeGadaaakeaacuWG2bGDgaqcamaaDaaaleaacqaIXaqmaeaacqaIZaWmcqaIWaamaaaaaa@3130@ higher and lower than median v^1
 MathType@MTEF@5@5@+=feaafiart1ev1aaatCvAUfKttLearuWrP9MDH5MBPbIqV92AaeXatLxBI9gBaebbnrfifHhDYfgasaacH8akY=wiFfYdH8Gipec8Eeeu0xXdbba9frFj0=OqFfea0dXdd9vqai=hGuQ8kuc9pgc9s8qqaq=dirpe0xb9q8qiLsFr0=vr0=vr0dc8meaabaqaciaacaGaaeqabaqabeGadaaakeaacuWG2bGDgaqcamaaBaaaleaacqaIXaqmaeqaaaaa@2F4D@, respectively. Log-rank-p-value= 0.000127. B) The curves represent the predicted survival for patients obtained using different values of *v1 *(estimated using the first 1500 genes), i.e., the 10^th ^(0.044), 25^th ^(0.215), 50^th ^(0.280), 75^th ^(0.428), and 90^th ^(0.563) percentiles of ***v1***.

DNA microarray technology allows for the development of predictive models using gene expression profiles to study the relationship between prognosis and molecular features of the tumor. New statistical methods have been developed to address high dimensionality and low sample size issues characterizing microarray data [21,23,24]. To predict survival in this group of melanoma patients, we applied SPC, a survival prediction model that was designed and validated using a leave-one-out cross-validation procedure [21]. Specifically, the model has been constructed applying the singular value decomposition procedure to the matrix *X' *consisting of only those genes whose Cox scores are greater than some threshold *θ *whose optimal value has been determined through a leave-one-out cross-validation procedure. In details, starting from a set G of possible values of *θ*, for each *θ *in G, one sample is left out, and those genes with absolute Cox scores greater than *θ *in the other n-1 samples are used to calculate V^
 MathType@MTEF@5@5@+=feaafiart1ev1aaatCvAUfKttLearuWrP9MDH5MBPbIqV92AaeXatLxBI9gBaebbnrfifHhDYfgasaacH8akY=wiFfYdH8Gipec8Eeeu0xXdbba9frFj0=OqFfea0dXdd9vqai=hGuQ8kuc9pgc9s8qqaq=dirpe0xb9q8qiLsFr0=vr0=vr0dc8meaabaqaciaacaGaaeqabaqabeGadaaakeaacuWGwbGvgaqcaaaa@2DF1@ for the left out sample. A Cox proportional hazards model is then fit to v^1
 MathType@MTEF@5@5@+=feaafiart1ev1aaatCvAUfKttLearuWrP9MDH5MBPbIqV92AaeXatLxBI9gBaebbnrfifHhDYfgasaacH8akY=wiFfYdH8Gipec8Eeeu0xXdbba9frFj0=OqFfea0dXdd9vqai=hGuQ8kuc9pgc9s8qqaq=dirpe0xb9q8qiLsFr0=vr0=vr0dc8meaabaqaciaacaGaaeqabaqabeGadaaakeaacuWG2bGDgaqcamaaBaaaleaacqaIXaqmaeqaaaaa@2F4D@ (i.e., to the first supervised principal component of V^
 MathType@MTEF@5@5@+=feaafiart1ev1aaatCvAUfKttLearuWrP9MDH5MBPbIqV92AaeXatLxBI9gBaebbnrfifHhDYfgasaacH8akY=wiFfYdH8Gipec8Eeeu0xXdbba9frFj0=OqFfea0dXdd9vqai=hGuQ8kuc9pgc9s8qqaq=dirpe0xb9q8qiLsFr0=vr0=vr0dc8meaabaqaciaacaGaaeqabaqabeGadaaakeaacuWGwbGvgaqcaaaa@2DF1@) and the chi-square statistic for the log-rank test associated with this model calculated. The procedure is repeated for each value of *θ *and any sample in the dataset. The overall likelihood ratio test indicated that the association between left-out samples and the supervised predictor constructed on v^1
 MathType@MTEF@5@5@+=feaafiart1ev1aaatCvAUfKttLearuWrP9MDH5MBPbIqV92AaeXatLxBI9gBaebbnrfifHhDYfgasaacH8akY=wiFfYdH8Gipec8Eeeu0xXdbba9frFj0=OqFfea0dXdd9vqai=hGuQ8kuc9pgc9s8qqaq=dirpe0xb9q8qiLsFr0=vr0=vr0dc8meaabaqaciaacaGaaeqabaqabeGadaaakeaacuWG2bGDgaqcamaaBaaaleaacqaIXaqmaeqaaaaa@2F4D@ is optimized at a value of *θ *= 1.7 at a p-value = 0.0002 (Supplementary Fig. S1). Thus, the survival prediction model has been constructed using the first principal component of those genes having a Cox score greater than 1.7 and resulted in 30 survival-related probes (Fig. 1, in black the transcripts used to build the SPC survival predictor).

Fig. 2, panel A illustrates the survival probability of the 43 samples, grouped according to the median value of v^130
 MathType@MTEF@5@5@+=feaafiart1ev1aaatCvAUfKttLearuWrP9MDH5MBPbIqV92AaeXatLxBI9gBaebbnrfifHhDYfgasaacH8akY=wiFfYdH8Gipec8Eeeu0xXdbba9frFj0=OqFfea0dXdd9vqai=hGuQ8kuc9pgc9s8qqaq=dirpe0xb9q8qiLsFr0=vr0=vr0dc8meaabaqaciaacaGaaeqabaqabeGadaaakeaacuWG2bGDgaqcamaaDaaaleaacqaIXaqmaeaacqaIZaWmcqaIWaamaaaaaa@3130@ = v^1
 MathType@MTEF@5@5@+=feaafiart1ev1aaatCvAUfKttLearuWrP9MDH5MBPbIqV92AaeXatLxBI9gBaebbnrfifHhDYfgasaacH8akY=wiFfYdH8Gipec8Eeeu0xXdbba9frFj0=OqFfea0dXdd9vqai=hGuQ8kuc9pgc9s8qqaq=dirpe0xb9q8qiLsFr0=vr0=vr0dc8meaabaqaciaacaGaaeqabaqabeGadaaakeaacuWG2bGDgaqcamaaBaaaleaacqaIXaqmaeqaaaaa@2F4D@ (*θ *= 1.7), i.e., the predicted values obtained selecting the first 30 probes corresponding to the threshold *θ *= 1.7, with short and long survivors having a value of v^130
 MathType@MTEF@5@5@+=feaafiart1ev1aaatCvAUfKttLearuWrP9MDH5MBPbIqV92AaeXatLxBI9gBaebbnrfifHhDYfgasaacH8akY=wiFfYdH8Gipec8Eeeu0xXdbba9frFj0=OqFfea0dXdd9vqai=hGuQ8kuc9pgc9s8qqaq=dirpe0xb9q8qiLsFr0=vr0=vr0dc8meaabaqaciaacaGaaeqabaqabeGadaaakeaacuWG2bGDgaqcamaaDaaaleaacqaIXaqmaeaacqaIZaWmcqaIWaamaaaaaa@3130@ higher and lower than the median, respectively.

Although these 30 predicting probes are all among the 80 transcript identified by SAM as associated to survival (Fig. 1), it has to be noted that class predictors constructed using a small number of variables may heavily depend on any one variable and produce spuriously high prediction strengths. However, the cross-validated predictor can also be constructed using a large number of transcripts without loosing statistical significance. Indeed, the overall likelihood ratio test indicates that the association between left-out samples and supervised predictor constructed on v^1
 MathType@MTEF@5@5@+=feaafiart1ev1aaatCvAUfKttLearuWrP9MDH5MBPbIqV92AaeXatLxBI9gBaebbnrfifHhDYfgasaacH8akY=wiFfYdH8Gipec8Eeeu0xXdbba9frFj0=OqFfea0dXdd9vqai=hGuQ8kuc9pgc9s8qqaq=dirpe0xb9q8qiLsFr0=vr0=vr0dc8meaabaqaciaacaGaaeqabaqabeGadaaakeaacuWG2bGDgaqcamaaBaaaleaacqaIXaqmaeqaaaaa@2F4D@ is still significant (p-value < 5%) for lower values of the threshold *θ *. As an examples, selecting a value of *θ *= 0.6 resulted in 1500 predicting probes which partitioned the samples according to the predicted survival curves of Fig. 2, panel B (v^11500
 MathType@MTEF@5@5@+=feaafiart1ev1aaatCvAUfKttLearuWrP9MDH5MBPbIqV92AaeXatLxBI9gBaebbnrfifHhDYfgasaacH8akY=wiFfYdH8Gipec8Eeeu0xXdbba9frFj0=OqFfea0dXdd9vqai=hGuQ8kuc9pgc9s8qqaq=dirpe0xb9q8qiLsFr0=vr0=vr0dc8meaabaqaciaacaGaaeqabaqabeGadaaakeaacuWG2bGDgaqcamaaDaaaleaacqaIXaqmaeaacqaIXaqmcqaI1aqncqaIWaamcqaIWaamaaaaaa@3312@ = v^1
 MathType@MTEF@5@5@+=feaafiart1ev1aaatCvAUfKttLearuWrP9MDH5MBPbIqV92AaeXatLxBI9gBaebbnrfifHhDYfgasaacH8akY=wiFfYdH8Gipec8Eeeu0xXdbba9frFj0=OqFfea0dXdd9vqai=hGuQ8kuc9pgc9s8qqaq=dirpe0xb9q8qiLsFr0=vr0=vr0dc8meaabaqaciaacaGaaeqabaqabeGadaaakeaacuWG2bGDgaqcamaaBaaaleaacqaIXaqmaeqaaaaa@2F4D@ (*θ *= 0.6)).

To adjust for prognostic factors, we also applied a multidimensional Cox model where the predicted value of the first principal component obtained by SPC using the first 30 and 1,500 probes (e.g., v^130
 MathType@MTEF@5@5@+=feaafiart1ev1aaatCvAUfKttLearuWrP9MDH5MBPbIqV92AaeXatLxBI9gBaebbnrfifHhDYfgasaacH8akY=wiFfYdH8Gipec8Eeeu0xXdbba9frFj0=OqFfea0dXdd9vqai=hGuQ8kuc9pgc9s8qqaq=dirpe0xb9q8qiLsFr0=vr0=vr0dc8meaabaqaciaacaGaaeqabaqabeGadaaakeaacuWG2bGDgaqcamaaDaaaleaacqaIXaqmaeaacqaIZaWmcqaIWaamaaaaaa@3130@ and v^11500
 MathType@MTEF@5@5@+=feaafiart1ev1aaatCvAUfKttLearuWrP9MDH5MBPbIqV92AaeXatLxBI9gBaebbnrfifHhDYfgasaacH8akY=wiFfYdH8Gipec8Eeeu0xXdbba9frFj0=OqFfea0dXdd9vqai=hGuQ8kuc9pgc9s8qqaq=dirpe0xb9q8qiLsFr0=vr0=vr0dc8meaabaqaciaacaGaaeqabaqabeGadaaakeaacuWG2bGDgaqcamaaDaaaleaacqaIXaqmaeaacqaIXaqmcqaI1aqncqaIWaamcqaIWaamaaaaaa@3312@) has been analyzed together with other prognostic factors, such as LDH plasma level and the number of metastases. Results from this multidimensional analysis indicate that the transcriptional patterns are significantly associated with survival time (p-value = 0.02 for v^130
 MathType@MTEF@5@5@+=feaafiart1ev1aaatCvAUfKttLearuWrP9MDH5MBPbIqV92AaeXatLxBI9gBaebbnrfifHhDYfgasaacH8akY=wiFfYdH8Gipec8Eeeu0xXdbba9frFj0=OqFfea0dXdd9vqai=hGuQ8kuc9pgc9s8qqaq=dirpe0xb9q8qiLsFr0=vr0=vr0dc8meaabaqaciaacaGaaeqabaqabeGadaaakeaacuWG2bGDgaqcamaaDaaaleaacqaIXaqmaeaacqaIZaWmcqaIWaamaaaaaa@3130@ and p-value = 0.06 for v^11500
 MathType@MTEF@5@5@+=feaafiart1ev1aaatCvAUfKttLearuWrP9MDH5MBPbIqV92AaeXatLxBI9gBaebbnrfifHhDYfgasaacH8akY=wiFfYdH8Gipec8Eeeu0xXdbba9frFj0=OqFfea0dXdd9vqai=hGuQ8kuc9pgc9s8qqaq=dirpe0xb9q8qiLsFr0=vr0=vr0dc8meaabaqaciaacaGaaeqabaqabeGadaaakeaacuWG2bGDgaqcamaaDaaaleaacqaIXaqmaeaacqaIXaqmcqaI1aqncqaIWaamcqaIWaamaaaaaa@3312@) also in the presence of other prognostic factors. On the contrary, LDH and the number of metastasis did not result statistically correlated with survival (p-value = 0.16 and p-value = 0.21 for LDH and p-value = 0.87 and p-value = 0.91 for number of metastasis considering v^130
 MathType@MTEF@5@5@+=feaafiart1ev1aaatCvAUfKttLearuWrP9MDH5MBPbIqV92AaeXatLxBI9gBaebbnrfifHhDYfgasaacH8akY=wiFfYdH8Gipec8Eeeu0xXdbba9frFj0=OqFfea0dXdd9vqai=hGuQ8kuc9pgc9s8qqaq=dirpe0xb9q8qiLsFr0=vr0=vr0dc8meaabaqaciaacaGaaeqabaqabeGadaaakeaacuWG2bGDgaqcamaaDaaaleaacqaIXaqmaeaacqaIZaWmcqaIWaamaaaaaa@3130@ and v^11500
 MathType@MTEF@5@5@+=feaafiart1ev1aaatCvAUfKttLearuWrP9MDH5MBPbIqV92AaeXatLxBI9gBaebbnrfifHhDYfgasaacH8akY=wiFfYdH8Gipec8Eeeu0xXdbba9frFj0=OqFfea0dXdd9vqai=hGuQ8kuc9pgc9s8qqaq=dirpe0xb9q8qiLsFr0=vr0=vr0dc8meaabaqaciaacaGaaeqabaqabeGadaaakeaacuWG2bGDgaqcamaaDaaaleaacqaIXaqmaeaacqaIXaqmcqaI1aqncqaIWaamcqaIWaamaaaaaa@3312@, respectively).

### Validation of microarray data

We validated at the protein level by immunohistochemistry (IHC) the results obtained from gene expression analysis. Of the first 80 probes, 5 coded for HLA class II molecules, which are constitutively expressed on antigen presenting cells and B cells, on non-immune cell types under inflammatory conditions and by melanoma cells [25,26]. Although we selected for microarray analysis melanoma biopsies that had a high percentage of tumor cells, the contribution of genes expressed at low level by cells present in tumor infiltrate cannot be ruled out. Moreover, 30 of 43 biopsies were infiltrated metastatic lymph nodes and thus a small amount of residual lymphoid tissue, expressing HLA class II molecules, could have been present.

**Figure 3:**
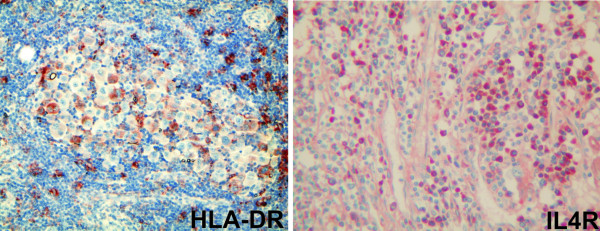
**Immunohistochemical analysis**. Representative sections of a melanoma biopsy immunostained with anti-HLA-DR (left panel, 20×), and anti-IL4R mAbs (right panel, 20×).

To assess if the expression of HLA class II molecules correlating with longer survival was due to melanoma cells or to other cells in the infiltrate, we verified by IHC the expression of these proteins with an antibody directed against HLA-DR molecules. Staining was performed in 33 of 43 melanoma biopsies for which corresponding paraffin-embedded specimens were available. HLA-DR expression was found on melanoma cells in 7 of 33 samples (Fig. 3 and Table 4), mainly with focal staining, while in the majority of cases (30/33) positivity was detected in the inflammatory cells that infiltrated melanoma deposits. On the other hand, a closer analysis of the two patient groups with significantly different survival lengths (as identified by SAM analysis) disclosed that HLA-DR expression was higher in melanoma cells of patients with longer survival than in those with shorter survival time (6 samples vs 1 sample, Table 4), while it was almost equally represented in the inflammatory cells of the two groups of patients.

**Table 4:** HLA-DR and IL4R expression determined by IHC as graded by the scoring system (1+ to 3+).

**Antigen**	**Patients with shorter survival**					**Patients with longer survival**				
	
	*Scoring*				*# of patients*	*Scoring*				*# of patients*
				
	*0*	*1+*	*2+*	*3+*		*0*	*1+*	*2+*	*3+*	
**HLA-DR**					**17**					**16**
tumor cells	16	1	0	0		10	5	0	1	
non tumor cells*	2	2	10	3		1	6	5	4	
**IL4R**					**14**					**13**
tumor cells	9	3	2	0		7	5	0	1	
non tumor cells*	13	1	0	0		8	3	2	0	

* lymphoid tissue and inflammatory interstitial cells

Up-regulation of HLA class II molecules can be induced by a number of molecules, including IL-4. This study demonstrated that increased expression of the IL-4R alpha chain gene was correlated with longer survival (Table 2). To confirm this finding, we investigated the presence of this protein in all available tumor biopsies by IHC. In 11 of 27 lesions, tumor cells expressed IL-4R (Fig. 3), while in 6 lesions a heterogeneous positive staining was observed also on inflammatory cells permeating the neoplasia (Table 4). Combining the staining on tumor and infiltrating cells, a higher incidence of IL-4R positive lesions was observed in patients with longer survival, therefore confirming the findings of the transcriptional analysis (Table 4).

CD4 and CD8 staining also revealed the presence of a mild to moderate T cell infiltrate inside melanoma cell nests in the majority of cases; usually intratumoral CD4^+ ^and CD8^+ ^T cells were co-localized within the lesion (data not shown).

### Association of the selected genes with survival

Eighty probes identified by SAM consisted of 40 genes whose increased expression was associated with longer survival, and 30 genes whose increased expression was associated with shorter survival. Genes involved in immune response and inflammation such as HLA class II, TRA@, LTB, TNFAIP3, IL-4R, IGLL1, CD1D, CD2, ITK, SOD2, DAF and GZMK, were associated selectively with longer survival, confirming once more the important interplay between the host immune system and malignant cells in melanoma.

Conversely, analysis of tumor progression-associated genes revealed an increased expression of those implicated in cell adhesion, motility and proliferation. For example, gene GJB2, encoding the gap junction protein connexin 26 (Cx26) a sub-type of gap junction proteins; gene CSPG4, coding for the melanoma-associated chondroitin sulfate proteoglycan 4; the disintegrin-like and metalloprotease genes with thrombospondin type 1 (ADAMTS5); the minichromosome maintenance 3 (MCM3) and the DCT genes, all whose expression by tumor cells is frequently associated with more invasive and metastatic behavior [27-33].

## Discussion

In this study, we used gene expression profiling and survival data from advanced melanoma patients and, by SAM class comparison, identified a set of 80 probes, corresponding to 70 genes, associated with survival. The survival-associated group of genes highlighted characteristics of melanoma cells, but also of non-malignant cells present in the tumor tissue. In fact, while specimens were selected on the basis of histological diagnosis, and characterized by a high proportion of neoplastic tissue, the use of whole tumor tissue enabled us to detect changes in gene expression associated with stromal and infiltrating immune/inflammatory cells.

Five of the probes selected using SAM encode HLA class II molecules. This MHC class II signature has already been associated with a favorable outcome in patients with large B-cell lymphoma [11], while in the liver microenvironment HLA class II overexpression is associated with a metastasis-inclined phenotype [34], suggesting that the prognostic genes may differ among different tumor types. In human melanoma, the association of these molecules with survival is controversial. Some authors reported that enhanced HLA class II expression was associated with an unfavorable prognosis [25,35], while others found a positive correlation with overall survival [36,37]. Our data are in line with the latter findings and the IHC analysis confirmed that the expression of these molecules in the melanoma cells was almost exclusively associated with longer survival (Table 4). CD4^+ ^and CD8^+ ^cells were found in the same lesions, and, as reported by others, a close correlation between MHC class II expression and presence of T cells was observed (data not shown). This phenomenon may be the result of an inflammatory process at the tumor site in which the cytokines released, such as IFN and TNF, induce MHC class II up-regulation. IL-4 may also be produced in an inflammatory milieu, and can up-regulate the expression of these molecules [38]. It has already been shown that melanoma cells may express IL-4 receptors and that IL-4 has antiproliferative and/or apoptotic effects in these cells [39]. Interestingly, the gene for the IL-4R alpha chain was included in the longer-survival set of genes, suggesting that its presence might be related to these cytotoxic pathways. As predicted by SAM, IHC revealed a positive staining for IL-4R, confirming at the protein level that enhanced expression was more evident in patients with longer survival (Table 4). IHC studies indicated also that IL-4R staining was localized in melanoma cells, as well as in interstitial inflammatory cells and in residual lymphoid tissue. Despite the intrinsic limitations of this study, these findings highlight the role of CD4^+ ^T-lymphocytes in the anti-melanoma immune response. Interestingly, the CD4 probe was among the first 225 probes selected by SAM, while the probe for CD8 was not included in the list of the first 3,000 differentially expressed genes.

The role of the immune system in survival of metastatic melanoma patients is also evident when other genes are considered. In addition to the MHC class II genes, other genes associated with survival direct the anti-tumor activity exerted by effector cells of the immune system such as T cells (TRA@, GZMK), NKT cells (CD1d) and B cells (IGLC2), confirming the notion that the clinical course of melanoma is closely associated with innate and acquired immune responses. It is of interest that genes known to be involved in the immune response were present exclusively in the longer-survival group, thus confirming an immune-response signature associated with favorable prognosis. The finding that genes associated with immune cell activation are part of the molecular signature that herald a good prognosis in stage III and IV melanoma patients is remarkable, since it highlights, at a molecular level, the potential efficacy of the immune response also in advanced melanoma. Thus, melanoma survival is associated not only with the intrinsic biology of tumor cells, but also with an efficient immune response that encompasses different facets of the immune system.

Of the genes correlated with shorter survival, some were already known to be over-expressed in different types of cancer, such as MCM3, BCHE [32,40-42], and some have previously been implicated in melanoma progression, like CSPG4 [43] and Cx26 [28] or, more generally, associated with tumor invasiveness, like ADAMTS5 [31].

The survival prediction model, designed using SPC, identified 30 survival-related probes, which were all among the 80 probes identified by SAM. Although we have not yet tested this predictive model on an independent series of patients, the results of the cross-validation were quite encouraging. Identifying a subset of genes that predict cancer patients' survival is an important goal of microarray research since it could ultimately lead to the development of prognostic tools that, by identifying patients with different clinical outcome, might lead to tailor therapeutic strategies on a single patient basis. This might be particularly relevant for patients with stage III melanoma, as the lack of definitively convincing results on interferon-alpha based adjuvant treatment might depend upon inadequate patient selection [44].

## Conclusion

In conclusion, this study applied high-throughput gene microarrays to screen the transcriptome in the search for genes correlated with patient survival. The bioinformatics data analysis allowed us to identify a number of genes related to cell proliferation and invasiveness that enable tumor cells to progress to a more aggressive phenotype. If validated by subsequent independent studies, these genes might serve as predictors of survival, and thus help clinicians to identify patients at higher risk of disease progression. Moreover, the transcripts associated to the longer-survival group included genes expressed in immune cells, both innate and acquired, confirming the importance of the interplay between immunological surveillance and the natural history of melanoma. These results highlight how critical the relationship is between melanoma cell aggressiveness and the activity of innate and/or acquired immune cells for the ultimate tumor outcome.

## Competing interests

The author(s) declare that they have no competing interests.

## Authors' contributions

SM conceived the study, carried out the molecular studies, coordinated the experiments and drafted the manuscript. GT and SF carried out the real-time PCR experiments and immunohistochemical analysis. MCM performed the histology analysis and supervised the immunohistochemical experiments. VCS was responsible for patients selection and clinical follow-up. SM,,CRR, EW and FMM participated in the design of the study. AC and SB partecipated in the design of the study and performed the statistical analysis. PZ partecipated in the design of the study and drafted the manuscript. All authors read and approved the final manuscript.

## Supplementary Material

Additional file 1 Calculation of survival prediction using supervised principal components. The data provided represent a detailed description of statistical methods used to calculate survival prediction. Click here for file
