# Supplementary material for: A gene expression signature associated with survival in metastatic melanoma
Source: J Transl Med. 2006 Nov 27;4:50. doi: 10.1186/1479-5876-4-50 (PMC1697826; doi:10.1186/1479-5876-4-50)
Supplement: Additional file 1 — Calculation of survival prediction using supervised principal components. The data provided represent a detailed description of statistical methods used to calculate survival prediction. [file 1479-5876-4-50-S1.doc]

**Additional files**

**Additional file 1 – Survival prediction calculation using supervised principal components.**

Supposing *X* a$pxn$ matrix of expression values (i.e., *p* genes and *n* patients) and$x_{ij}$ the expression level of the *i*th gene in the *j*th patient, the singular value decomposition of *X*:

$X=UDV^{T}$ Eq. (1)

defines *U*, a $pxn$ orthogonal matrix, *D*, a $nxn$ diagonal matrix, and *V*, a $nxn$ orthogonal matrix (Horn and Johnson 1985). The matrix V can be written as:

$V=X^{T}UD^{-1}$ Eq. (2)

where each row of *V* is a linear combination of the expression values in the corresponding column of *X.* Hence, the first few columns of V can be used as continuous predictors of survival for each patient. Formally:

$\hat{V}=X^{T}UD^{-1}$ Eq. (3)

Moreover, suppose that $\tilde{X}$is an independent test set, then

Eq. (4)

where *U* and *D* are the same as in Eq. (3) (i.e., derived from the singular value decomposition of the training data). In this case, the first few columns of $\hat{V}$ can be used to estimate the survival of patients in the independent test set. The rationale for choosing the first few columns of *V* is that the matrix *U* has been determined so that $X^{T}u_{1}$ has the largest sample variance amongst all normalized linear combinations of the rows of X. Hence, assuming that variations in gene expression accounts for variations in survival, $X^{T}u_{1}$ would capture a large percentage of the variation in survival. In theory, *V* could be calculated using the entire dataset *X*, and the rows of *V* would have different distributions depending on the survival time of the corresponding patient. In practice, however, many of the genes in *X* are unrelated to survival, and using the entire dataset *X* to compute *V*, will result in lowering the predictor quality. This problem can be solved using only genes with the largest Cox scores.


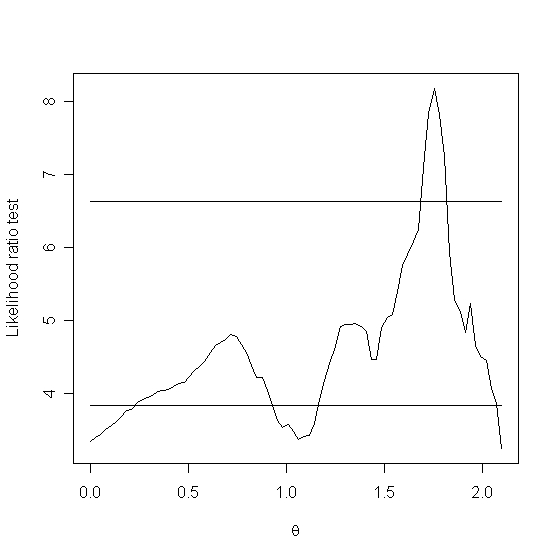


**Figure 1.** Likelihood ratio test of $\hat{v}_{1}$as a function of the threshold $\theta$ in the leave-one-out cross-validation procedure. Lower and upper bounds represents 5% and 1% confidence limits, respectively.
